# Episymbiotic Saccharibacteria induce intracellular lipid droplet production in their host bacteria

**DOI:** 10.1093/ismejo/wrad034

**Published:** 2024-01-10

**Authors:** Pu-Ting Dong, Jing Tian, Koseki J Kobayashi-Kirschvink, Lujia Cen, Jeffrey S McLean, Batbileg Bor, Wenyuan Shi, Xuesong He

**Affiliations:** Department of Microbiology, The ADA Forsyth Institute, Boston, MA 02142, United States; Department of Oral Medicine, Infection, and Immunity, Harvard School of Dental Medicine, Boston, MA 02115, United States; Department of Pediatric Dentistry, Peking University School and Hospital of Stomatology, Beijing 100081, China; Klarman Cell Observatory, Broad Institute of MIT and Harvard, Cambridge, MA 02142, United States; Laser Biomedical Research Center, G. R. Harrison Spectroscopy Laboratory, Massachusetts Institute of Technology, Cambridge, MA 02139, United States; Department of Microbiology, The ADA Forsyth Institute, Boston, MA 02142, United States; Department of Periodontics, University of Washington, Seattle, WA 98195, United States; Department of Microbiology, The ADA Forsyth Institute, Boston, MA 02142, United States; Department of Oral Medicine, Infection, and Immunity, Harvard School of Dental Medicine, Boston, MA 02115, United States; Department of Microbiology, The ADA Forsyth Institute, Boston, MA 02142, United States; Department of Microbiology, The ADA Forsyth Institute, Boston, MA 02142, United States; Department of Oral Medicine, Infection, and Immunity, Harvard School of Dental Medicine, Boston, MA 02115, United States

**Keywords:** Saccharibacteria, lipid droplets, episymbiosis, interspecies interaction, Candidate Phyla Radiation

## Abstract

Saccharibacteria (formerly TM7) are a group of widespread and genetically diverse ultrasmall bacteria with highly reduced genomes that belong to Candidate Phyla Radiation, a large monophyletic lineage with poorly understood biology. *Nanosynbacter lyticus* type strain TM7x is the first Saccharibacteria member isolated from the human oral microbiome. With restrained metabolic capacities, TM7x lives on the surface of, and forms an obligate episymbiotic relationship with its bacterial host, *Schaalia odontolytica* strain XH001. The symbiosis allows TM7x to propagate but presents a burden to host bacteria by inducing stress response. Here, we employed super-resolution fluorescence imaging to investigate the physical association between TM7x and XH001. We showed that the binding with TM7x led to a substantial alteration in the membrane fluidity of XH001. We also revealed the formation of intracellular lipid droplets in XH001 when forming episymbiosis with TM7x, a feature that has not been reported in oral bacteria. The TM7x-induced lipid droplets accumulation in XH001 was confirmed by label-free Raman spectroscopy, which also unveiled additional phenotypical features when XH001 cells are physically associated with TM7x. Further exploration through culturing XH001 under various stress conditions showed that lipid droplets accumulation was a general response to stress. A survival assay demonstrated that the presence of lipid droplets plays a protective role in XH001, enhancing its survival under adverse conditions. In conclusion, our study sheds new light on the intricate interaction between Saccharibacteria and their host bacteria, highlighting the potential benefit conferred by TM7x to its host and further emphasizing the context-dependent nature of symbiotic relationships.

## Introduction

The recently discovered Candidate Phyla Radiation (CPR) contributes to around 26% of bacterial diversity with potentially 73 new phyla [1]. Among these phyla, Saccharibacteria are a group of widespread and genetically diverse ultrasmall bacteria with reduced genomes [2]. Ever since its discovery based on 16S rRNA gene sequencing in 1996 [3], Saccharibacteria have been detected in a wide range of natural habitats, from the soil, and deep-sea sediments to various human body sites, including the gastrointestinal tract, genital tract, and skin [4–9]. Saccharibacteria are particularly prevalent in the oral cavity though with low abundance [10]. However, its relative abundance increases (as high as over 20% of the total oral bacterial community as reported in some studies) in patients with various types of periodontitis [8, 11]. Research also demonstrated the plausible correlation between Saccharibacteria and the occurrence of lung cancer [12]. Like other CPR bacteria, Saccharibacteria are known for their recalcitrance to cultivation with limited cultivated strains [2]. As the first cultivated representative of Saccharibacteria, “*Nanosynbacter lyticus*” type strain TM7x HMT-952 was isolated from the human oral cavity, and this enabled the discovery of its parasitic behavior with its bacterial host *Schaalia odontolytica* (formerly *Actinomyces odontolyticus*) strain XH001 [13, 14].

TM7x is ultrasmall in size (200–300 nm) with a highly reduced genome (705 kb) and lacks amino acid and nucleotide biosynthetic capability [13]. It is an obligate epibiont parasite living on the surface of and inducing stress in its bacterial host cell XH001 [15, 16]. A recent study demonstrated that, through a complete arginine deiminase system, an arginine catabolism pathway acquired during its environment-to-mammal niche transition, TM7x can catabolize arginine, produce adenosine triphosphate and ammonia, then confers a beneficial effect toward its host cells XH001, especially in the low-pH environment [14]. A comprehensive transcriptome study from TM7x and XH001 provides mechanistic insights into this episymbiotic lifestyle in the level of gene expression [17].

In this study, we utilized super-resolution fluorescence microscopy to offer nanoscale insights into the physical interactions between TM7x and XH001. We found that the infection of TM7x had a significant impact on the membrane fluidity of host cells XH001, resulting in a distinct distribution of liquid-ordered and disordered phases compared to the organized distribution observed in XH001 cells alone. Additionally, we discovered the enhanced production of intracellular lipid droplets (LDs) in the XH001 cells when physically associated with TM7x. Furthermore, we employed label-free noninvasive Raman spectroscopy to capture multiple phenotypic differences based on the Raman spectra, demonstrating saturated fatty acids, a main component of LDs, being the most prominent contributor to these signature differences. We also showed that XH001 cells alone exhibited the accumulation of saturated fatty acids when subjected to various stress conditions, indicating that LD formation could be a general response to stress and serves as a stress marker. The subsequent starvation assay further demonstrated that the accumulation of fatty acids likely plays a beneficial role in protecting XH001 cells against stress factors and enhancing their survival. Overall, the combination of advanced imaging techniques and label-free Raman spectroscopy revealed some of the emergent features in XH001 when forming symbiosis with TM7x and provided valuable new insights into this intriguing bacterial interspecies interaction.

## Results

### Super-resolution fluorescence imaging of BODIPY C_1_, C_12_-labeled XH001 and XH001/TM7x

Previous studies have shown that TM7x predominantly resides on the surface of its host bacterium XH001 through fluorescence *in situ* hybridization (FISH) imaging [13, 15] and phase contrast microscopy. However, the morphology of TM7x remains difficult to resolve as the size of the TM7x cells is around 200–300 nm, close to the diffraction limit [18]. Microscopic approaches with subdiffraction limit capacity are needed. Through super-resolution fluorescence imaging achieved by confocal fluorescence microscopy (Materials and methods) with an Airyscan detector [19], a lateral resolution of 120 nm can be achieved [20]. To visualize both TM7x and host cells XH001, we utilized a fluorescence dye, BODIPY C_1_, C_12_ (Materials and methods), which labels every organelle that is hydrophobic [21]. We compared the image quality of BODIPY C_1_, C_12_-stained XH001/TM7x under the conventional confocal microscope and confocal microscope with Airyscan detector, respectively. Fluorescence images under a confocal microscope with Airyscan detector apparently exhibit higher image resolution. Moreover, BODIPY can label both XH001 cells and TM7x cells (Supplementary Fig. 1).

**Figure 1:**
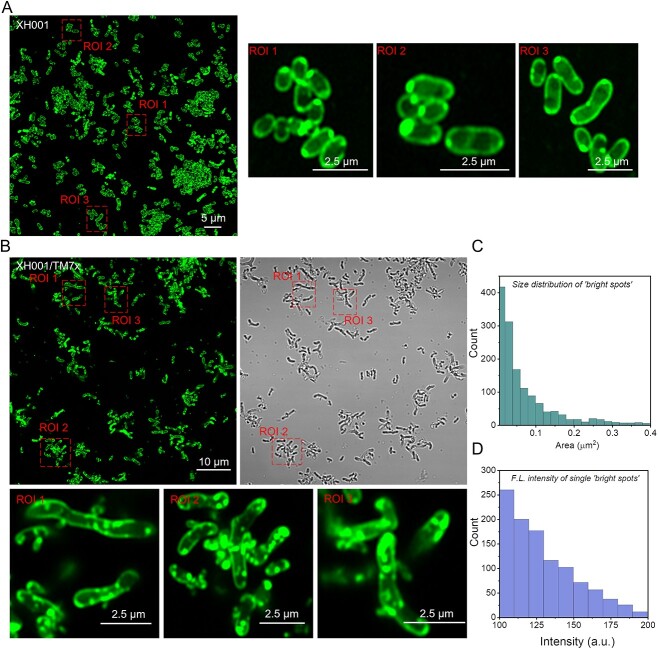
Confocal laser scanning imaging of BODIPY C_1_, C_12_-labeled monoculture XH001 cells and coculture XH001/TM7x with Airyscan detector; (A and B) super-resolution fluorescence imaging of host cells XH001 and XH001/TM7x along with zoom-in views of three regions of interest (ROIs), respectively; (C) size distribution of “bright dots” from (B); (D) fluorescence intensity distribution of individual “bright spots” from (C); fluorescence images were obtained after labeling XH001 or XH001/TM7x with BODIPY for 30 min and sandwiched between a cover glass and cover slide; scalar bars are labeled directly on the corresponding images.

We then compared the morphology of BODIPY C_1_, C_12_-labeled monoculture XH001 cells, and XH001/TM7x coculture under the confocal microscope with Airyscan detector. Monoculture XH001 cells displayed fluorescence signals mainly from the cell membrane and some from bright dots that were mostly associated with the membrane (Fig. 1A), presenting a rod-shaped morphology. However, in the XH001/TM7x coculture, XH001 cell morphology is highly irregular, and fluorescence signals are from both the cell membrane and mostly intracellular “bright dots” (Fig. 1B). These “bright dots” showed an average area of 0.099 μm^2^ corresponding to a 340-nm diameter dot size (Fig. 1C). And they demonstrated excellent fluorescence intensity (Fig. 1D) compared to the fluorescence stain from the cell membrane. It was also known that BODIPY C_1_, C_12_ has been widely employed to visualize neutral lipids or LDs [22, 23]. We thus suspected that these intracellular “bright dots” might be LDs.

### Laurdan fluorescence imaging suggests the host cell membrane fluidity change induced by TM7x

One of the characteristic features of episymbiosis is the intimate physical interaction between epibiont and its host cell. To further understand the cell membrane physiological change in host bacterium XH001 induced by TM7x, Laurdan was employed to characterize the membrane fluidity and organization. Laurdan, a membrane fluorophore sensitive to the local membrane packing, has been extensively utilized to quantify the degree of lipid packing and membrane fluidity due to the dipolar relaxation effect [24, 25]. Under an excitation wavelength of 405 nm, the emission spectrum of Laurdan peaked at 490 nm when the membrane lipids are in a disordered phase (liquid-disordered phase, more fluid) and shifted to ~440 nm when the membrane lipids are in a more packed situation (liquid-ordered phase, more rigid) [26–28]. Then a generalized polarization (GP) value can be derived to quantify the membrane fluidity:


$$ \mathrm{GP}=\frac{I_{\mathrm{Lo}}-{I}_{\mathrm{Ld}}}{I_{\mathrm{Lo}}+{I}_{\mathrm{Ld}}}, $$

where ${I}_{\mathrm{Lo}}$ and ${I}_{\mathrm{Ld}}$ are the fluorescence intensities from liquid-ordered phase and liquid-disordered phase, respectively. Therefore, the obtained GP values range from −1 (being least ordered, very fluid) to +1 (being most ordered, very rigid).

**Figure 2:**
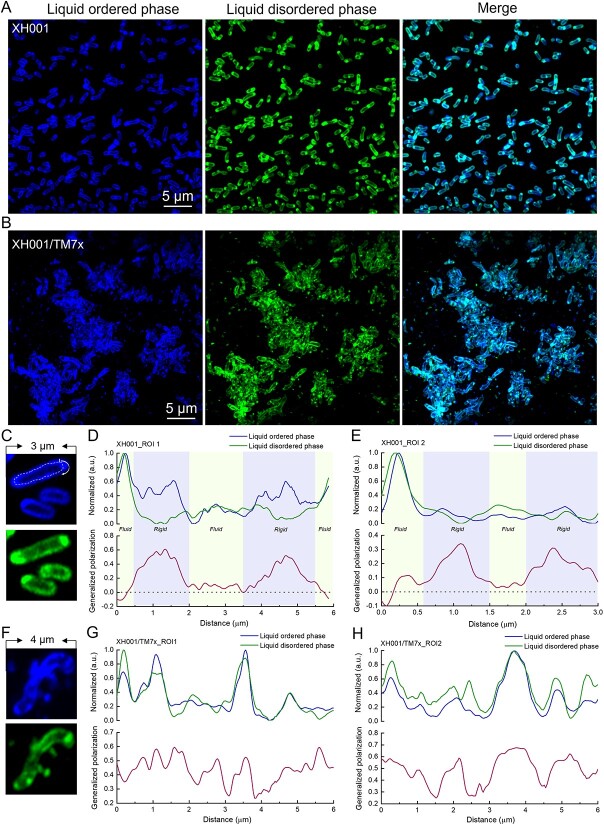
Mapping membrane fluidity of XH001 and XH001/TM7x through super-resolution fluorescence imaging of Laurdan-labeled XH001 and XH001/TM7x; (A and B) super-resolution fluorescence images of liquid-ordered phases and liquid-disordered phases from XH001/TM7x and XH001, respectively; excitation laser: 405 nm; fluorescence signal from the liquid-ordered/disordered phase was collected by an optical bandpass filter (410–460 nm) and (470 nm–550 nm), respectively; scalar bar = 5 μm; (C–E) zoom-in view of Laurdan-labeled XH001 cells along with single-cell mapping of GP; (F–H) zoom-in view of Laurdan-labeled XH001/TM7x along with single-cell mapping of GP; a GP value of 0 was highlighted by black dashed lines; panels (C–H) are representative of at least 10 cells randomly chosen from multiple fields of view.

To image and quantify membrane fluidity at the nanometer scale, super-resolution fluorescence imaging with an Airyscan detector was employed to harvest the fluorescence images from liquid-disordered phase and liquid-ordered phase by choosing corresponding emission filters (Materials and methods). The liquid-ordered phase fluorescence signal of XH001 cells is mainly from the parallel edges, whereas liquid-disordered phase fluorescence is mostly from the distal ends of cells (Fig. 2A). By contrast, when forming symbiosis with TM7x, XH001 cells displayed more randomly distributed fluorescence signals from two channels, and the membrane patches of liquid-ordered/disordered phase seem to be stochastically allocated (Fig. 2B).

GP values of XH001 cells from monoculture and XH001/TM7x coculture were then obtained based on the above equation. Here, to better understand the distribution of lipid patches from liquid-ordered and -disordered phases along the cell membrane, circular GP values of the host cell membrane were calculated starting from the distal end of the cell (Fig. 2C). The central parts of XH001 cells are more rigid with a GP value of around 0.3–0.6. The distal ends of host cells exhibit smaller GP values (0–0.1), indicative of fluid lipids clustered together at the ends of XH001 cells (Fig. 2D and E). This evidence suggests that lipid packing in XH001 cells from monoculture is highly oriented. On the other hand, XH001 cells forming symbiosis with TM7x displayed a highly randomized lipid packing (Fig. 2F–H) as GP value has no specific oscillation pattern along the cell peripheral. These data suggest that symbiosis with TM7x drastically affects the organization of lipid molecules in XH001 cell membrane. In short, through Laurdan fluorescence imaging, we showed that membrane fluidity of host cells XH001 is significantly altered as a result of TM7x infection: once ordered lipid packing becomes highly randomized. Meanwhile, as a hydrophobic dye, Laurdan staining also revealed intracellular LD-like structures in XH001 when forming symbiosis with TM7x.

### LipidSpot staining consolidates the formation of intracellular lipid droplets in XH001 when forming symbiosis with TM7x

**Figure 3:**
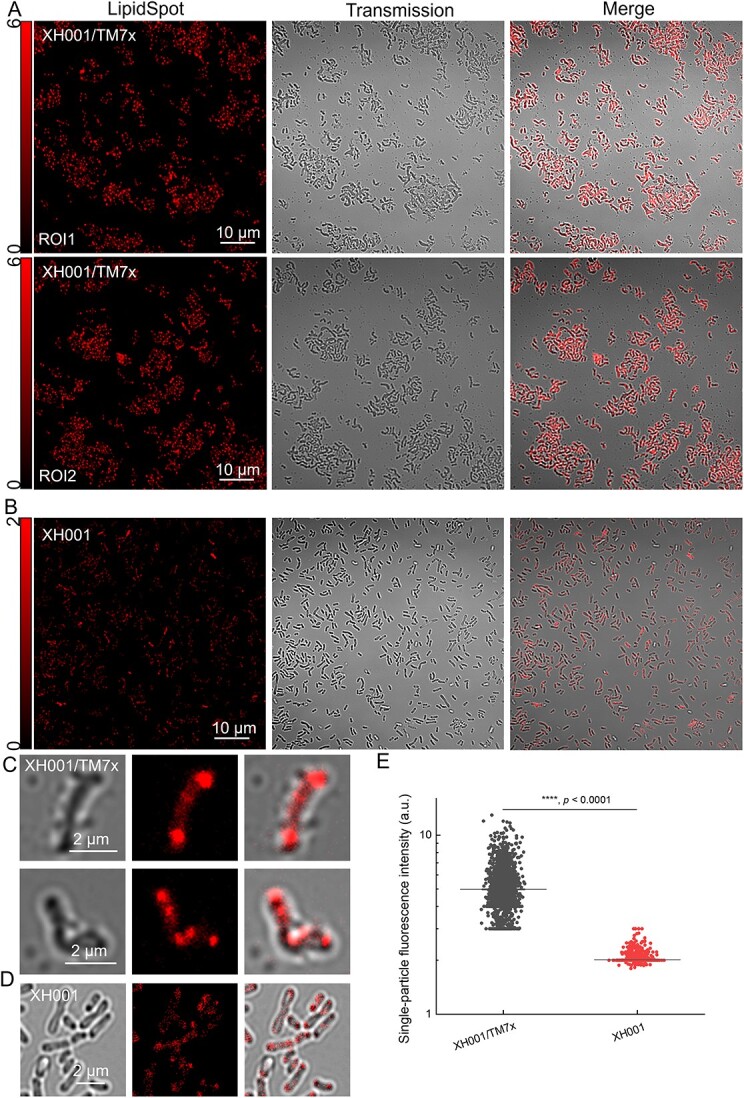
Confocal laser scanning imaging of LipidSpot-labeled XH001/TM7x and XH001; (A and B) fluorescence images, transmission images, and merged images of LipidSpot-labeled XH001/TM7x from two ROIs and XH001 cells, respectively; scalar bar = 10 μm; (C and D) zoom-in view of LipidSpot-labeled XH001/TM7x and XH001 cells, respectively; (E) quantification of single-droplet fluorescence intensity from monoculture XH001 and coculture XH001/TM7x; the data were collected from at least 2000 individual droplets within each group; statistical analysis was determined through Student’s unpaired *t*-test: ^*^^*^^*^^*^, *P* < .0001.

LD, a monolayer phospholipid membrane-bound organelle found in almost all eukaryotes, plays essential roles in cellular lipid homeostasis [29]. However, bacterial intracellular LDs are less common and remain underexplored. Meanwhile, the “bright dots” as revealed by BODIPY staining resemble membrane vesicles physically [30, 31]. Thus, to confirm these BODIPY and Laurdan-stained “bright spots” are indeed LDs, we utilized an LDs-specific dye, ‘LipidSpot [32]’, to stain XH001 and XH001/TM7x (Materials and methods). Confirming our previous staining, we observed lots of LDs showing up in the coculture XH001/TM7x from different fields of view (Fig. 3A). On the contrary, very faint signals can be detected in XH001 cells from monoculture (Fig. 3B), especially under different color bars. A higher magnification view further consolidates this phenomenon (Fig. 3C and D). Moreover, all of these “LDs” are localized intracellularly at the cell poles or are associated with the cell membrane. And, TM7x alone was not labeled by this dye. Quantitative analysis also demonstrates that fluorescence intensity from individual LD in the case of XH001/TM7x is significantly higher than that of monoculture XH001 (Fig. 3E). To investigate the spatial arrangement of LDs in relationship to TM7x attachment, we also counterstained TM7x using FISH in the LipidSpot-stained coculture XH001/TM7x. TM7x closely correlates with LDs (with a pair correlation value >1, Supplementary Fig. 2), which suggests LDs tend to form in the area that is spatially close to the associated TM7x cells. Collectively, data from BODIPY- and Laurdan-stained images as well as LipidSpot-labeled fluorescence images strongly indicated that the episymbiotic interaction with TM7x significantly induces the production of intracellular LDs.

### Raman spectroscopy reveals the enhanced production of lipid droplets in XH001 when associated with TM7x

BODIPY C_1_, C_12_, Laurdan, and LD-specific fluorescence imaging collectively indicated an increase in LD production within XH001 cells when associated with TM7x. To further validate these observations, we sought to explore alternative methodologies, such as Raman spectroscopy, to acquire independent confirmation. Moreover, this noninvasive assay provides a comprehensive perspective on the shift in cellular metabolites within XH001 cells upon interaction with TM7x.

Raman spectroscopy has the capability to probe the chemical compositions of biological analytes in a label-free and noninvasive manner [33–36]. Raman scattering is a process of inelastic scattering of photons by molecular bonds, given that every chemical bond inside a specific molecule has distinctive vibrational energy. We thus wondered how different these intracellular biomolecules might be when XH001 are TM7x-free versus attached to TM7x. To answer this question, cells from XH001 monoculture and XH001/TM7x coculture were fixed inside 10% formalin, washed with milli-Q water, and then air-dried onto an aluminum substrate. Raman spectra of XH001 cells and XH001/TM7x were then acquired under a HORIBA Raman spectrometer (Materials and methods).

**Figure 4:**
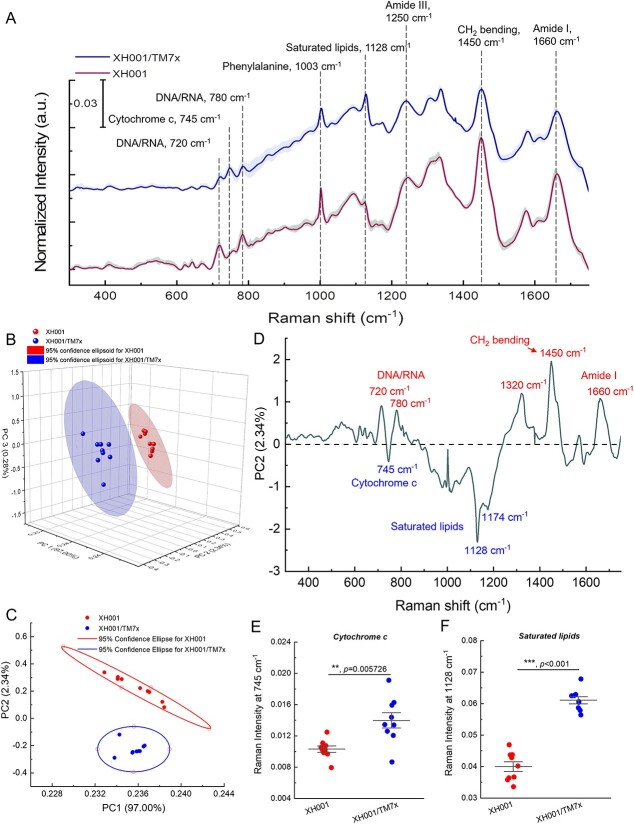
Raman spectroscopy of XH001 and XH001/TM7x along with the corresponding PCA; (A) Raman spectra of XH001 cells and XH001/TM7x from three biological replicates after vector normalization; data: mean (solid line) ± standard deviation (SD); interested Raman peaks were highlighted by dashed lines; (B) 3D PCA (PCA) plot of Raman spectra of XH001 cells and XH001/TM7x; each dot represents a Raman spectrum; data are from three biological replicates; (C) 2D PCA plot from principal components 1 (97.00% of the total variation) and 2 (2.34% of the total variation); 95% confidence intervals were represented by ellipses; (D) spectral information of PC2; biomolecules with enhanced production in the case of XH001/TM7x were highlighted below the dashed line and XH001 cells above the dashed line; (E and F) quantitative and statistical analyses of Raman intensities from cytochrome c (745 cm^−1^) and saturated lipids (1128 cm^−1^); statistical analysis was conducted through a two-tailed Student’s unpaired *t*-test; ^*^^*^^*^: *P* < .001.

The fingerprint region spanning from 320 to 1800 cm^−1^ effectively captures the majority of vibrational data related to intracellular biomolecules, revealing the distinctive and observable Raman phenotype characteristic of cells (Fig. 4A and Supplementary Fig. 3). Following vector normalization (Materials and methods), the Raman spectra of both XH001 and XH001/TM7x cells yielded the identification of several typical Raman peaks: 720 cm^−1^ (DNA or RNA), 780 cm^−1^ (DNA or RNA), 745 cm^−1^ (cytochrome c), 1003 cm^−1^ (phenylalanine), 1128 cm^−1^ (saturated lipids) [37], and 1660 cm^−1^/1250 cm^−1^ (Amide I/III, protein). The Raman intensities of certain peaks, such as cytochrome c, exhibited significant disparities. These variations cannot be solely attributed to the additive presence of TM7x (Supplementary Fig. 3).

To provide a comprehensive representation of the Raman spectral distinctions, we employed principal component analysis (PCA). PCA, a powerful statistical technique, enables the reduction of data dimensions while retaining the majority of the variance present in the original dataset [38]. Subsequently, we generated 2D and 3D plots, utilizing different combinations of scores from the first two or three components, respectively. In the context of the 3D plot (Fig. 4B), a divergence emerges between the Raman spectra of XH001 in monoculture and those of XH001/TM7x. The 2D PCA plot (Fig. 4C), based on the first two PCs, demonstrates a substantial and statistically significant disparity in the PC2 scores between XH001 and XH001/TM7x (*P* < .001, Student’s unpaired *t*-test).

To decipher which specific biomolecules within PC2 contribute significantly to the discernible distinctions in the Raman spectra between XH001 monoculture and XH001/TM7x coculture, we extracted the spectral information associated with PC2, responsible for elucidating 2.34% of the total variation. As depicted in Fig. 4D, Raman peaks at 745, 1128, and 1174 cm^−1^ stand out in the case of XH001/TM7x in comparison with XH001. These Raman peaks correspond to cytochrome c (745 cm^−1^) and saturated lipids (1128 and 1174 cm^−1^) [37, 39], respectively. Furthermore, the spectral details of PC2 unveil additional significant disparities encompassing nucleic acids (720/780 cm^−1^) and Amide I (1660 cm^−1^).

There was a difference not only in the pattern of the peaks but also in the intensity of the peaks. Therefore, we performed a quantification of intracellular biomolecules by integrating Raman bands at 745 (cytochrome c, Fig. 4E) and 1128 cm^−1^ (saturated fatty acids, Fig. 4F). This quantification approach is underpinned by the linear relationship between Raman intensity and the concentration of biomolecules [40]. We found that there is a significantly higher signal (*P* < .001) in the Raman spectra of XH001/TM7x compared to XH001 regarding the content of cytochrome c and saturated lipids. In particular, the augmented production of saturated fatty acids, which are primary constituents of LDs [41], from XH001/TM7x coculture is consistent with our abovementioned microscopic evidence.

### XH001 cells exhibit enhanced accumulation of lipid droplets in the presence of various stress factors

It has been demonstrated that prokaryotes possess the capacity to accumulate lipophilic compounds within the cytoplasm, forming lipid inclusions [41]. These lipophilic compounds, such as polyhydroxybutyrate, triacylglycerol, and wax ester, are accumulated in response to stress (e.g. low nitrogen availability) [42]. Previous studies [15, 17] indicated that the symbiosis with XH001 allows TM7x to propagate but often presents a burden to host bacteria by inducing stress response, particularly in the stable symbiosis state. We wondered whether the formation of LDs observed through fluorescence microscopy and Raman spectroscopy was due to stress-induced host response. To explore this phenomenon, we cultured XH001 cells under diverse stress conditions, including distinct oxygen levels (0% and 21%), which have been documented to elicit varying degrees of stress responses in XH001 cells [15]. We also subjected XH001 cells to treatments with various H_2_O_2_ concentrations.

**Figure 5:**
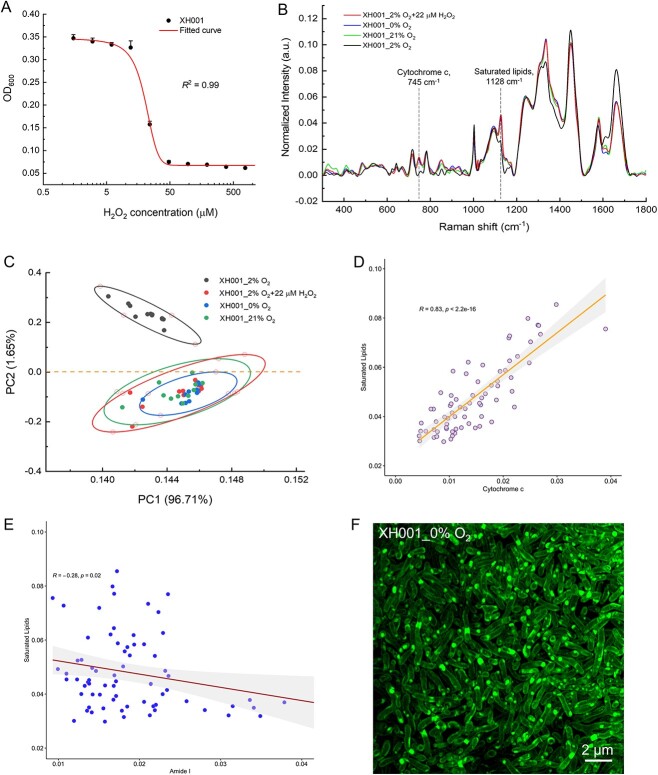
Characterization of XH001 cells under various stressed conditions by Raman spectroscopy and super-resolution fluorescence imaging; (A) MIC of H_2_O_2_ against host cells XH001 alone; data were fitted by a dose–response relationship function; (B) Raman spectra of XH001 cells cultured under various stressed conditions, with each spectrum averaged from at least 10 individual plots of three biological replicates; (C) 2D PCA plot of Raman spectra acquired from XH001 cells under different stressed conditions; plots were depicted from principal components 1 (96.71% of the total variation) and 2 (1.65% of the total variation); 95% confidence intervals were represented by ellipses; (D) correlation analysis between cytochrome c (745 cm^−1^) and saturated lipids (1128 cm^−1^); Spearman’s correlation coefficient and *P*-value were labeled in the plots; (E) correlation analysis between Amide I (1660 cm^−1^) and saturated lipids (1128 cm^−1^); Spearman’s correlation coefficient and *P*-value were labeled in the plots; (F) super-resolution fluorescence imaging of BODIPY C_1,_ C_12_-labeled XH001 cells cultured inside the anaerobic conditions; scalar bar = 2 μm.

As illustrated in Fig. 5A, the minimum inhibitory concentration (MIC) of H_2_O_2_ against XH001 cells is 44 μM. To maintain viability while inducing stress, we opted for a sub-MIC concentration of 22 μM to treat XH001 cells. In all, XH001 cells were treated with 22-μM H_2_O_2_, or grew under 0% O_2_, or 21% O_2_, and subsequently fixed in 10% formalin and analyzed by Raman spectra to assess potential phenotypic differences. Distinct Raman peaks at 745 cm^−1^ (cytochrome c), 1128 cm^−1^ (saturated fatty acids), and 1660 cm^−1^ (Amide I, protein) were evident in the groups subjected to different stress (Fig. 5B) compared to XH001 cells cultured under the optimized condition (2% O_2_). Global differences between XH001 cells cultured under optimized and stressed conditions were revealed through 2D PCA (Fig. 5C), with principal component 2 illustrating the most significant difference. All stress conditions clustered together with XH001/TM7x coculture compared to XH001 alone (Supplementary Fig. 4). Spearman’s correlation analysis also demonstrated that saturated lipids display a significant positive correlation with cytochrome c (Fig. 5D), and a negative correlation with Amide I (Fig. 5E). Additionally, we examined the formation of LDs using super-resolution fluorescence microscopy. Figure 5F displays BODIPY C_1_, C_12_-labeled XH001 cells under the anaerobic cultured condition and revealed apparent LD accumulation, especially when comparing XH001 cells cultured under the microaerophilic condition (Fig. 1B).

### Accumulation of lipid droplets enhances cell survival under stressed conditions

**Figure 6:**
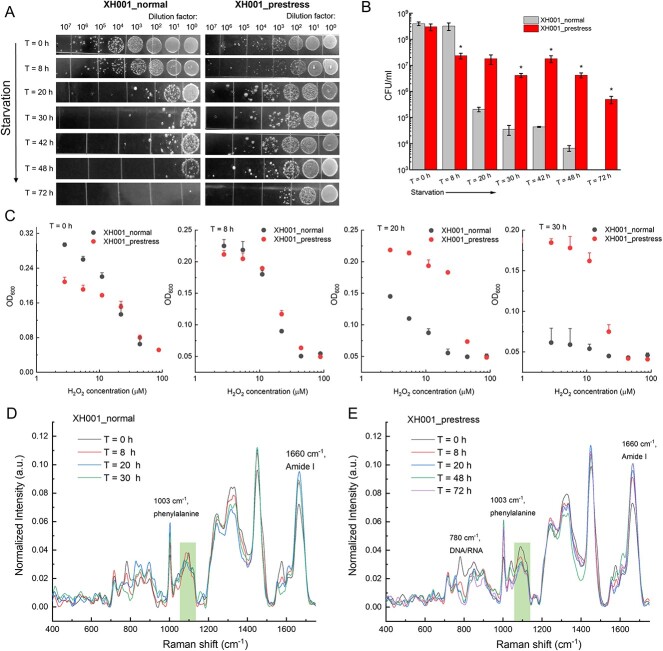
Saturated fatty acids potentially contribute to the survival of XH001 cells under stressed conditions; (A) CFUs of XH001 cells with and without prestress under time-course starvation conditions; (B) quantification analysis of CFU from (A); data: mean ± SD from three replicates; statistical analysis was conducted between the normal and prestressed groups by a Student’s unpaired *t*-test. ^*^: *P* < .05; (C) dose–response of H_2_O_2_ against XH001 cells under both normal and prestressed conditions at different starvation times; mean + SD from three biological replicates; (D and E) Raman spectra from XH001 cells under both normal (D) and prestressed conditions (E); data were averaged from at least 10 spectra acquired from three biological replicates; ROIs were highlighted by rectangular boxes.

To investigate the biological implications of increased accumulation of fatty acids, we conducted a starvation assay as follows (Materials and methods): XH001 cells cultured under both optimized (XH001_normal) and prestressed (0% O_2_, overnight) conditions (XH001_prestress) were extensively washed to remove the residual medium and resuspended in 1 × PBS and incubated in the optimal growth condition (2% O_2_). The temporal changes in colony-forming unit (CFU) assay, Raman spectra, and H_2_O_2_ susceptibility test were determined for each culture. The first drastic difference was observed in CFU where normal and prestressed XH001 groups began to display separation at 20 h post starvation (Fig. 6A and B). XH001_normal group suffered significantly more (three orders of magnitude) reduction in CFU compared to the prestress group at 48 h poststarvation, suggesting that prestress enhances the survival of XH001 cells under starvation conditions. Moreover, the H_2_O_2_ susceptibility test between the two groups provided further evidence that prestress significantly boosts XH001 cells’ resilience against oxidative environments (Fig. 6C). Consistently, Raman spectroscopy analysis demonstrated that the amount of saturated lipids decreases as the starvation process endures in the group XH001_prestress (Fig. 6D and E), and this is positively correlated with the time-course decrease in CFU of prestressed XH001 cells, which further consolidates the protective role of saturated fatty acids. We also noted the change in the intracellular amount of other biomolecules (Supplementary Fig. 5), such as amino acids (1660 cm^−1^) and nucleic acids (DNA or RNA, 780 cm^−1^), which suggests fatty acid accumulation may not be the only factor contributing to the cell survival under stressed conditions.

### Accumulation of lipid droplets under stressed conditions is not a general feature of human-associated bacteria

We then wondered whether the accumulation of saturated fatty acids and the formation of intracellular LDs when under stress are general features of human-associated bacteria. We examined the characteristics of other bacteria, such as *Fusobacterium nucleatum* (*Fn*), *Streptococcus mutans* (*S. mutans* UA159) as well as a pathogenic *Staphylococcus aureus* strain, methicillin-resistant *S. aureus* (MRSA) USA300, in the presence of sub-MIC H_2_O_2_ by Raman spectroscopy and super-resolution fluorescence microscopy.

**Figure 7:**
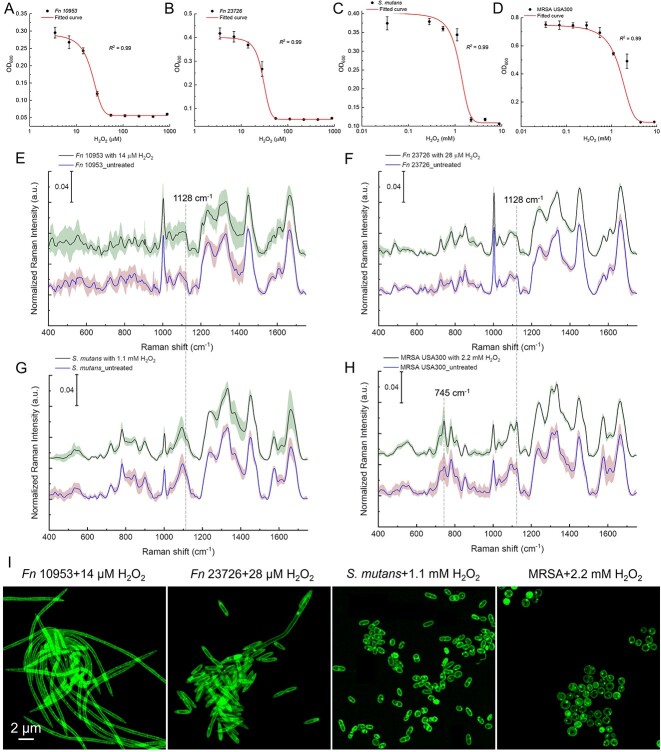
Characterization of other bacteria under stress conditions by Raman spectroscopy and super-resolution fluorescence microscopy; (A–D) dose–response of H_2_O_2_ against *Fn* 10 953, *Fn* 23 726, *S. Mutans*, and MRSA USA300; data were fitted by a dose–response relationship function; (E–H) Raman spectra of *Fn* 10 953 (E), *Fn* 23 726 (F), *S. mutans* (G), and MRSA USA300 (H) with and without the treatment of H_2_O_2_; data: mean (solid line) ± SD (shadow) from three biological replicates; Raman peaks at 745 and 1128 cm^−1^ were highlighted by dashed lines; (I) super-resolution fluorescence imaging of BODIPY C_1_, C_12_-labeled *Fn* 10 953, *Fn* 23 726, *S. Mutans*, and MRSA after the treatment of sub-MIC H_2_O_2_; scalar bar = 2 μm.

We assessed the H_2_O_2_ susceptibility of *Fn* ATCC 10953, *Fn* ATCC 23726, *S. mutans*, and MRSA USA300 (Fig. 7A–D). Subsequently, we selected a sub-MIC concentration of H_2_O_2_ to induce stress in the four bacteria. Raman spectra were then acquired from fixed bacteria, both with and without the addition of H_2_O_2_. Unlike XH001 cells under stress, those bacteria do not exhibit increased Raman intensity at 1128 cm^−1^, indicating a lack of accumulation of saturated fatty acids (Fig. 7E–H).

In the case of MRSA USA300 with and without the treatment of sub-MIC H_2_O_2_, we noticed an increase with regard to Raman intensity at 745 and 1128 cm^−1^ (Fig. 7H). It has been reported that both saturated lipids and cytochrome c contribute to the Raman peak at 1128 cm^−1^ [39, 43, 44]. To discern the impact of cytochrome c upon the Raman intensity at 1128 cm^−1^, we then quantified the integral Raman intensity of cytochrome c and saturated lipids from both XH001 and MRSA cells under stressed conditions. As illustrated in Supplementary Fig. 6, a notable 3-fold difference emerged between cytochrome c and saturated lipids in the context of XH001 cells subjected to stressful conditions (*P* < .0001). By contrast, no significant distinction was discerned for MRSA under stress conditions (*P* = .06). This observation strongly suggested that, in the case of MRSA, the Raman intensity at 1128 cm^−1^ predominantly originated from cytochrome c, as indicated by the pronounced Raman intensity at 745 cm^−1^. Moreover, the super-resolution BODIPY C_1_, C_12_ imaging analysis revealed the absence of discernible LD formation in these bacteria when exposed to stressful conditions. Hence, this compelling evidence hints at the notion that the accumulation of saturated fatty acids, as we observed in XH001, may not present a general feature in oral bacteria.

## Discussion

Illustrating the physical interaction between Saccharibacteria and their host bacteria is essential to understanding this intimate, yet understudied interspecies interaction. Conventional microscopes have limited resolution to clearly depict this episymbiotic relationship. Here, through super-resolution fluorescence imaging, the morphology of coculture XH001/TM7x was clearly discernible with around 120-nm lateral resolution under BODIPY staining (Fig. 1). Rod-shaped host cells XH001 become irregular in the XH001/TM7x coculture, which might be related to the lower expression of chromosomal partitioning genes [17]. Laurdan quantification indicated that the cell membrane fluidity or lipid packing of host cells XH001 was drastically affected due to the association with TM7x: from highly ordered to randomly distributed (Fig. 2). We discovered the formation of intracellular LDs in XH001 cells in the coculture (Figs 1 and 2). LipidSpot assay and label-free Raman spectroscopy assay further consolidated this discovery (Figs 3 and 4). The data imply that epibiont Saccharibacteria significantly alter the host cell membrane organization and elicit the cellular metabolic response, leading to the accumulation of saturated fatty acids and the enhanced formation of LDs.

LDs are a unique multifunctional organelle comprising a phospholipid monolayer surrounding a hydrophobic neutral lipid core that contains cholesterol esters, triacylglycerols, and wax esters [45]. They are ubiquitous lipid storage organelles existing in all eukaryotic cells [29, 46], especially when the cells are under stress or challenge. Intracellular LDs are less common in bacteria, with limited reports. Gram-positive actinobacteria and Cyanobacteria, for example, such as *Mycobacterium*, *Streptomyces*, and *Rhodococcus*, contain wax esters inside LDs, especially under stress or nutrition-depleted environment conditions [47]. A recent study identified the existence of LDs in gut bacteria, and these LDs might be closely linked to human health [42]. Our data showed that XH001 cells display enhanced saturated fatty acid production and formation of intracellular LDs when exposed to various stress conditions (Fig. 5). Meanwhile, the increase in intracellular LDs is positively associated with XH001’s enhanced tolerance to starvation and oxidative stress (Fig. 6). A recent study demonstrated that bacterial LDs bind to DNA via intermediary protein(s), thus protecting DNA and enhancing survival when exposed to genotoxic stressors [48]. The improved resistance of XH001 to hydrogen peroxide (Fig. 6), a genotoxic stressor, could potentially reflect the protective role of LDs in maintaining DNA stability, which is worth further examination. Thus, the enhanced accumulation of LDs could be a general response of XH001 to stress, which allows bacteria to better cope with adverse conditions for better survival. Our previous studies showed that the TM7x association reduced the XH001 growth rate and triggered the expression of stress-related genes [17]. Therefore, the enhanced formation of LDs could be XH001’s response to TM7x-induced stress, but this may provide a survival advantage for both XH001 and TM7x under stressful conditions, thus entailing an important ecological consequence.

Intracellular bacterial parasite infections often lead to the accumulation of host intracellular LDs, which are used by parasites for nutrients [49] or immune evasion [50]. Thus, our finding also prompted the speculation that the observed TM7x-triggered LDs accumulation in XH001 could be a more specific parasite–bacterial host interaction. Genomic analysis reveals that CPR organisms, including Saccharibacteria*,* lack the capacity for synthesizing membrane lipids required for the cell envelope [1] while possessing membrane lipids rich in fatty acids [51]. TM7x may induce LD production in XH001 to meet its growth demand. The observation that XH001 intracellular LDs tended to form in the area spatially close to the associated TM7x cells (Supplementary Fig. 2) seems to be in line with this notion, as the close spatial proximity may facilitate the uptake of LD-incased lipids by TM7x through XH001/TM7x contact zone. Hence, we hypothesize that Saccharibacteria might symbiotically obtain lipids or lipids building blocks from host bacteria, a question that warrants further investigation.

Raman peaks at 1128 and 1174 cm^−1^ further suggest that the major lipid molecules inside those LDs might be saturated lipids such as polyhydroxybutyrate [52], myristic acid, and stearic acid [37]. For future work, mass spectrometry-based lipidomic analyses can be employed to determine which specific saturated lipid molecule accounts for the major component of these LDs. We also found that coculture XH001/TM7x has significantly higher cytochrome c content compared to host cells XH001 alone (Fig. 4E). And, the difference was not from the direct addition of TM7x as pure TM7x culture did not exhibit an apparent Raman intensity from those peaks (Supplementary Fig. 3). Cytochrome c, the ubiquitous heme-containing protein existing in all the life domains, is a key protein in the respiratory electron transfer chain of cells [53]. The higher content of cytochrome c suggests an enhanced respiration process for the host bacteria in the coculture XH001/TM7x. Further studies regarding the correlation between an enhanced respiratory activity with potential bacterial stress are needed to unravel this issue. Besides cytochrome c and saturated lipids, we also noticed other spectroscopic differences between XH001 cells and XH001/TM7x in Amide I/III (proteins, 1660/1250 cm^−1^). Raman intensities of Amide I/III peaks in XH001 cells are higher than that of XH001/TM7x cells, implying that TM7x association elicits a metabolic activity shift inside the host cells.

In this study, through super-resolution fluorescence imaging and label-free Raman spectroscopy, we revealed some new features of XH001 host cells induced by Saccharibacteria, such as changes in cell membrane fluidity and the enhanced formation of LDs in XH001 as a result of symbiotic interaction with TM7x. However, due to the ultrasmall size of TM7x and the still limited lateral resolution of Airyscan detector, future work is warranted to acquire nanoscale visualization of TM7x cell structures using advanced microscopic techniques such as stochastic optical reconstruction microscopy [18] or expansion microscopy [54] at nanoscale resolution. Another interesting direction to pursue is to find out the dominant lipid biomolecules that play an essential role in the attachment between TM7x and host cells. Taken together, our findings provide a new perspective to understand the evolutionary strategies of Saccharibacteria and their host bacteria.

## Materials and methods

### Bacterial strains and growth conditions

XH001 monoculture and XH001/TM7x coculture were isolated from the oral cavity as described in the previous published study [13]. Strains were cultured in brain heart infusion (BHI, Thermo Fisher Scientific, NH) at 37°C under different oxygen conditions as specified in the main text: anaerobic (0% O_2_, 10% CO_2_, 5% H_2_, balanced with N_2_), microaerophilic (2% O_2_, 5% CO_2_, balanced with N_2_), and atmospheric conditions (∼21% O_2_, 0.04% CO_2_, 0.9% Ar, 78% N_2_). To acquire growth kinetics and phase contrast images, three independent cell cultures were grown under the specified oxygen condition for two passages (1 ml culture inoculated into 10 ml BHI and incubated 24 h each) before being reinoculated into 20 ml fresh BHI. The optical density at 600 nm (OD_600_) was measured using a spectrophotometer (Thermo Fisher Scientific, MA).

*Fusobacterium nucleatum* strains were cultured in Columbia broth (Thermo Fisher Scientific, MA) at 37°C under anaerobic condition (0% O_2_, 10% CO_2_, 5% H_2_, balanced with N_2_) for two passages.

*Streptococcus mutans* UA159 and MRSA were cultured inside BHI broth at aerobic condition (∼21% O_2_, 0.04% CO_2_, 0.9% Ar, 78% N_2_) for two passages.

### Minimum inhibitory concentration of H_2_O_2_ against multiple bacteria

For XH001 cells, *F. nucleatum* ATCC23726, *F. nucleatum* ATCC10953, an initial bacterial inoculum with an OD_600_ of 0.1 was prepared and added to a sterile 96-well plate (Thermo Fisher Scientific, MA); 880 μM of H_2_O_2_ was added to the front row of a 96-well plate, then a 2-fold serial dilution was performed with a resulting concentration of 880, 440, 220 … 0 μM. After overnight incubation inside the microaerophilic chamber (XH001 cells) and anaerobic chamber (*F. nucleatum* cells), the OD_600_ of the 96-well plate was recorded by a plate reader (iMark™ microplate absorbance reader, Bio-Rad). The MIC was determined as the lowest concentration at which no visible growth occurs. Sub-MIC bacterial inoculum was fixed with 10% formalin and was washed three times with 1 × PBS for the Raman spectra acquisition and fluorescence imaging.

For *S. mutans* and MRSA, all steps mirrored the aforementioned procedure, with the exception that the bacterial inoculum featured a concentration of 1 × 10^5^ cells/ml, the initial H_2_O_2_ concentration stood at 8.8 mM, and the cells were incubated within an aerobic chamber.

### Starvation assay

XH001 cells were initially cultured in a microaerophilic chamber for two passages. Subsequently, two distinct groups were prepared: an untreated group and a prestress group. The cells in each group were diluted and cultured overnight, with the untreated group incubated inside a microaerophilic chamber and the prestress group within an anaerobic chamber. Following this, the cells were harvested, subjected to two rounds of washing with sterile 1 × PBS, and ultimately suspended in PBS within the microaerophilic chamber. Starvation happened over time, samples were collected at each specific point, and subjected to the following CFU assay, susceptibility test toward H_2_O_2,_ and Raman spectra acquisition.

### Fluorescence microscopy

For BODIPY C_1_, C_12_ fluorescence imaging, cells were fixed, washed with 1 × PBS, and stained with BODIPY C_1_, C_12_ (Thermo Fisher Scientific, MA) with a working concentration of 1.5 μM for 30 min. After that, cells were washed twice with 1 × PBS and were sandwiched below a cover glass (Thermo Fisher Scientific) and glass slide (Thermo Fisher Scientific, NH). Fluorescence images were acquired at an excitation wavelength of 488 nm and an emission window from 500–550 nm. Confocal laser scanning microscopy was conducted by a ZEISS LSM880 (Carl Zeiss AG, Germany) system with a 63× (NA = 1.4) oil immersion objective.

For LipidSpot fluorescence imaging, live cells (XH001 cells, XH001/TM7x cells) were stained with LipidSpot (Biotium) at a 1 × working concentration for 3–5 h. After that, cells were immediately collected to acquire the fluorescence images at an excitation wavelength of 488 nm and an emission window from 500 to 550 nm under a ZEISS LSM880 confocal laser scanning microscope.

For Laurdan-based membrane fluidity measurements, Laurdan (6-Dodecanoyl-2 Dimethylaminonaphthalene Thermo Fisher Scientific, MA) was dissolved in DMF (Sigma Aldrich, MA) and a final concentration of 1% DMF was maintained in the medium for better solubility. Cells were washed two times with prewarmed PBS and were further incubated with Laurdan for 30 min. After that, cells were washed two times with prewarmed PBS and were sandwiched between a cover glass and a cover slide. Laurdan fluorescence intensities were measured at 460 ± 5 and 500 ± 5 nm upon excitation at 405 nm under a ZEISS LSM880 confocal laser scanning microscope with the Airyscan detector. Images were acquired with a 63× (NA = 1.4) oil immersion objective. Laurdan GP value was calculated using the formula GP = (*I*_460_ − *I*_500_)/(*I*_460_ + *I*_500_).

Airyscan imaging was performed with a confocal laser scanning microscope ZEISS LSM 880 equipped with an Airyscan detection unit. To maximize the resolution enhancement, we used high NA oil immersion Plan-Apochromat 63× (NA = 1.40) Oil Corr M27 objectives (Zeiss, Germany). All imaging were performed using Immersol 518 F immersion media (*n_e_* = 1.518 (23°C); Carl Zeiss). Detector gain and pixel dwell times were adjusted for each dataset, keeping them at their lowest values in order to avoid saturation and bleaching effects.

For Airyscan processing (super-resolution fluorescence imaging), Zen Black software was used to process the acquired images. The software processes each of the 32 Airy detector channels separately by performing filtering, deconvolution, and pixel reassignment in order to obtain images with enhanced spatial resolution and improved signal-to-noise ratio. This processing includes a Wiener filter deconvolution with options of either a 2D or a 3D reconstruction algorithm (details are described elsewhere) [19]. In our analysis, we applied the Airyscan Processing Baseline Shift and further used the 2D or 3D reconstruction algorithm either at the default filter setting or at filter settings as noted.

All acquired images were analyzed through FiJi (NIH) and CellProfiler.

### Measurement and analysis of Raman spectra

Cultured cells were fixed in 10% formalin (neutral buffered, Sigma Aldrich) for 60 min and were washed three times with sterile water. Before Raman measurements, the fixed cells were dropped onto an aluminum-coated Raman substrate to be air-dried. Raman spectra were acquired using an HR Evolution confocal Raman microscope (Horiba Jobin-Yvon, France) equipped with a 532-nm neodymium-yttrium aluminum garnet laser. The laser power on cells was 8 mW after attenuation by neutral density filters (25%). An objective with a magnification of 100× (NA = 0.6) was used to focus single cells with a laser spot size of ∼1 μm^2^, and Raman scattering was detected by a charge-coupled device cooled at −70°C. The spectra were acquired in the range of 300–1800 cm^−1^ with 600 grooves per mm diffraction grating. The acquisition parameters were 10 s with two accumulations per spectrum, at least nine spectra from three biological replicates. All obtained Raman spectra were preprocessed by comic ray correction and polyline baseline fitting with LabSpec 6 (Horiba Scientific, USA). Spectral normalization was done by vector normalization of the entire spectral region. The selection of vector normalization was undertaken with the aim of rectifying general instrumentation fluctuations as well as mitigating the influence of sample and experimental variables (such as the thickness of the sample) while minimizing interference with the inherent characteristics of the biological content. Data analysis, statistics, and visualization were performed by OriginLab (OriginLab Corporation, MA) and RStudio 2023.03.0 + 386 environment using in-house scripts.

### Statistical analysis

Two-tailed Student’s unpaired *t*-test and one-way analysis of variance were used to determine whether there is any statistically significant difference between groups (^*^P < .05, ^*^^*^P < .01, ^*^^*^^*^P < .001, ^*^^*^^*^^*^P < .0001).

## Supplementary Material

Supplementary_Figures-12132023_wrad034

## Data Availability

All data needed to evaluate the conclusions in the paper are present in the paper and/or the Supplementary Materials. Additional data related to this paper may be requested from the authors.
