# Supplementary material for: Episymbiotic Saccharibacteria induce intracellular lipid droplet production in their host bacteria
Source: ISME J. 2024 Jan 10;18(1):wrad034. doi: 10.1093/ismejo/wrad034 (PMC10939385; doi:10.1093/ismejo/wrad034)
Supplement: Supplementary_Figures-12132023_wrad034 [file supplementary_figures-12132023_wrad034.docx]

**Episymbiotic Saccharibacteria induces intracellular lipid droplet production in their host bacteria**

Pu-Ting Dong, Jing Tian, Koseki J. Kobayashi-Kirschvink, Lujia Cen, Jeffrey S. McLean, Batbileg Bor, Wenyuan Shi, Xuesong He

**Supplementary Figures**

**Supplementary Figure 1. Comparison of the fluorescence images acquired under the conventional confocal microscope and confocal microscope with airyscan detector (super-resolution fluorescence imaging**). Labeled TM7x cells were indicated by red arrows.

**Supplementary Figure 2. Spatial arrangement between lipid droplets and TM7x (FISH staining) in the co-culture between XH001 and TM7x. A**. Confocal fluorescence images of BODIPY-labeled XH001/TM7x cells and TM7x (after fluorescence in situ hybridization). Scalar bar = 10 µm. **B**. Pairwise correlation from the above two channels. The highest pair correlation value was labeled at a dipole distance of 0.7 µm.

**Supplementary Figure 3. Averaged Raman spectra of XH001, co-culture XH001/TM7x with prophage xhp1, and TM7x. A**. Averaged Raman spectra of XH001, XH001/TM7x, and TM7x cells in the range of 200 to 3200 cm^-1^. Three different regions were highlighted with different colors (fingerprint region, cell silent region, and C-H region). B. Comparison of the Raman spectra in the C-H region from the above three cells. **C**. Lorentzian fitting of Raman spectrum in the C-H region of TM7x cells. Three major components were decomposed under a Lorentzian fitting.

**Supplementary Figure 4. Spectral information of principal component 2 which contributes to 1.65% of the total variation.** Peaks of interest were highlighted in the plot.

**Supplementary Figure 5. Principal component analysis of Raman spectra acquired from pre-stressed XH001 cells at different starvation times.** Plots were depicted from principal component 1 (93.20% of the total variation) and 2 (2.41% of the total variation). 95% confidence intervals were represented by ellipses. Peaks of interest were highlighted in the plot.


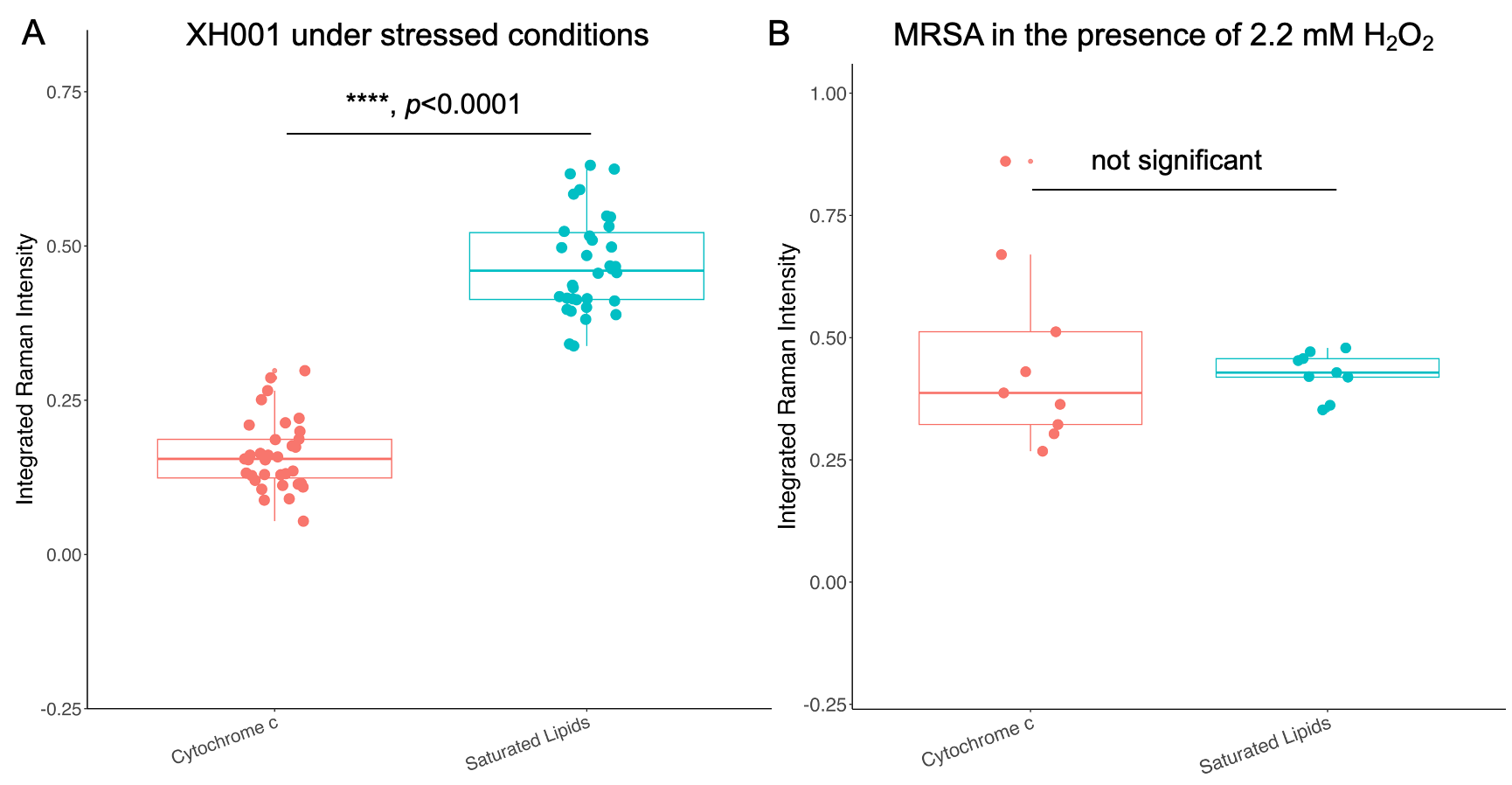


**Supplementary Figure 6. Quantification of the integrated Raman Intensity from cytochrome c and saturated lipids in the case of XH001 cells and *Staphylococcus aureus* cells under stressed conditions**. Each dot is from a single Raman spectrum. Statistical analysis was achieved by a two-tailed student unpaired *t*-test.
